# 3-Amino-*N*-benzyl-6-(4-fluoro­phen­yl)thieno[2,3-*b*]pyridine-2-carboxamide

**DOI:** 10.1107/S160053681202212X

**Published:** 2012-05-19

**Authors:** Jin-Ni Zhao, Sheng-Yong Yang, Li Yang

**Affiliations:** a State Key Laboratory of Biotherapy and Cancer Center, West China Hospital, West China Medical School, Sichuan University, Chengdu 610041, People’s Republic of China

## Abstract

In the title compound, C_21_H_16_FN_3_OS, the thieno[2,3-*b*]pyridine system forms dihedral angles of 10.57 (12) and 83.87 (5)° with the fluoro­phenyl ring at the 6-position and the phenyl ring of the benzyl group, respectively. In the crystal, mol­ecules are linked by weak N—H⋯N anf N—H⋯O hydrogen bonds and π–π stacking inter­actions involving fluoro­phenyl rings of adjacent mol­ecules, with a centroid–centroid distance of 3.648 (10) Å. In addition, intra­molecular N—H⋯S and N—H⋯O hydrogen bonds contribute to the stability of the mol­ecular conformation.

## Related literature

For the biological activity of thieno[2,3-*b*]pyridine derivatives, see: Litvinov *et al.* (2005 ▶).
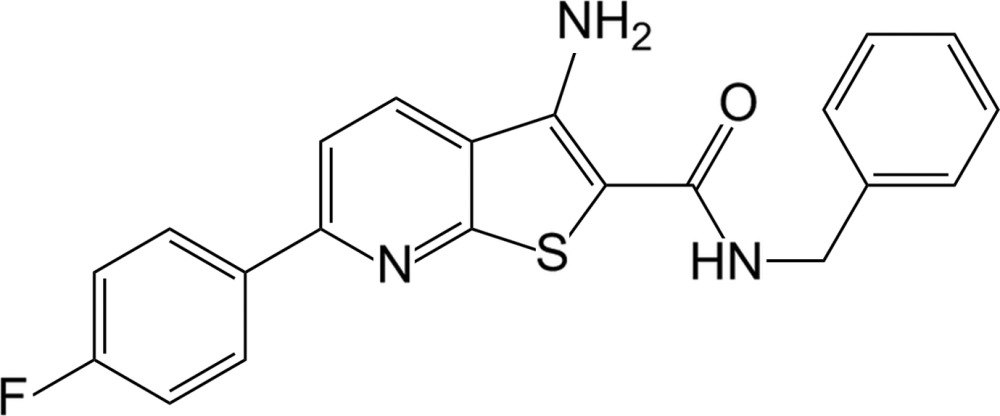


## Experimental

### 

#### Crystal data

C_21_H_16_FN_3_OS
*M* *_r_* = 377.43
Monoclinic, 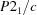

*a* = 18.9008 (7) Å
*b* = 9.9828 (4) Å
*c* = 9.5924 (4) Å
β = 102.224 (4)°
*V* = 1768.89 (11) Å^3^
*Z* = 4
Mo *K*α radiation
μ = 0.21 mm^−1^
*T* = 293 K
0.40 × 0.30 × 0.10 mm

#### Data collection

Oxford Diffraction Xcalibur Eos diffractometer
Absorption correction: multi-scan (*CrysAlis PRO*; Oxford Diffraction, 2006 ▶) *T*_min_ = 0.991, *T*_max_ = 1.000
7919 measured reflections
3609 independent reflections
2665 reflections with *I* > 2σ(*I*)
*R*_int_ = 0.021

#### Refinement

*R*[*F* ^2^ > 2σ(*F* ^2^)] = 0.044
*wR*(*F* ^2^) = 0.104
*S* = 1.03
3609 reflections
252 parameters
H atoms treated by a mixture of independent and constrained refinement
Δρ_max_ = 0.23 e Å^−3^
Δρ_min_ = −0.23 e Å^−3^

### 

Data collection: *CrysAlis PRO* (Oxford Diffraction, 2006 ▶); cell refinement: *CrysAlis PRO*; data reduction: *CrysAlis PRO*; program(s) used to solve structure: *SHELXS97* (Sheldrick, 2008 ▶); program(s) used to refine structure: *SHELXL97* (Sheldrick, 2008 ▶); molecular graphics: *OLEX2* (Dolomanov *et al.*, 2009 ▶) and *Mercury* (Macrae *et al.*, 2006 ▶); software used to prepare material for publication: *OLEX2*.

## Supplementary Material

Crystal structure: contains datablock(s) I, global. DOI: 10.1107/S160053681202212X/ds2191sup1.cif

Structure factors: contains datablock(s) I. DOI: 10.1107/S160053681202212X/ds2191Isup2.hkl

Supplementary material file. DOI: 10.1107/S160053681202212X/ds2191Isup3.cml

Additional supplementary materials:  crystallographic information; 3D view; checkCIF report

## Figures and Tables

**Table 1:** Hydrogen-bond geometry (Å, °)

*D*—H⋯*A*	*D*—H	H⋯*A*	*D*⋯*A*	*D*—H⋯*A*
N3—H3⋯S1	0.86	2.68	3.0897 (16)	111
N3—H3⋯N2^i^	0.86	2.51	3.253 (2)	146
N2—H2*B*⋯O1	0.90 (2)	2.15 (2)	2.741 (2)	122.7 (17)
N2—H2*B*⋯O1^ii^	0.90 (2)	2.20 (2)	3.015 (2)	150.0 (17)

Symmetry codes: (i) 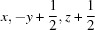; (ii) 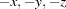.

**Fig. 1.**
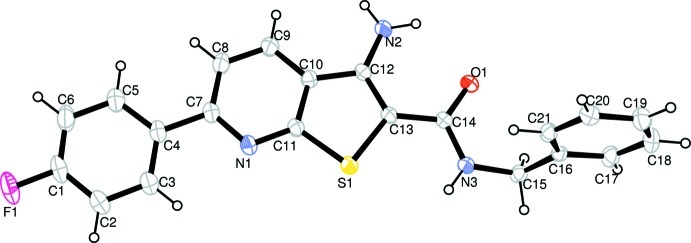
The molecular structure of the title compound, with displacement ellipsoids drawn at the 30% probability level.

**Fig. 2.**
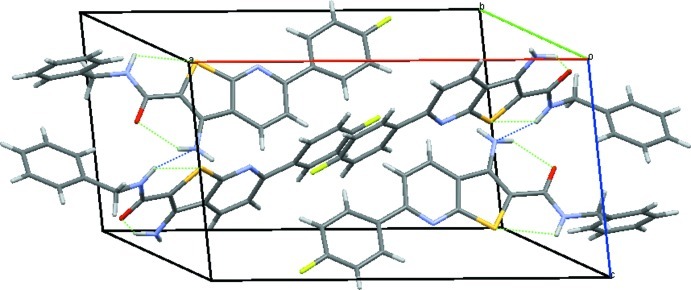
A packing diagram of the title compound, showing intermolecular hydrogen bonds of N—H···N as blue dashed lines,and intramolecular hydrogen bonds of N—H···O and N—H···S as green dashed lines

## supplementary crystallographic information

### Comment

Thieno[2,3-*b*]pyridine derivatives are of great importance owing to their wide biological properties (Litvinov *et al.*, 2005). The title compound is one of the key intermediates in our synthetic investigations of anticancer drugs. Herein we report its crystal structure.

As shown in Fig. 1, the thieno[2,3-*b*]pyridine ring forms dihedral angles of 10.57(0.12)° and 83.87(0.05)° with the monofluoro-benzene at 6-position and the phenyl rings at 2-position, respectively.In the crystal packing, stacking interactions involving phenyl rings containing fluorine atom of adjacent molecules are helpful for the stabilization of the crystal as well as intermolecular N—H···N hydrogen bonds.In addition,intramolecular N—H···O and N—H···S hydrogen bonds help to stabilize the molecular conformation (Table 1 and Fig.2).

### Experimental

To a solution of 6-(4-fluorophenyl)-2-thioxo-1,2-dihydropyridine-3-carbonitrile (2.30 g, 10 mmol) in DMF (15.00 ml) was added dropwise a solution of 10% sodium hydroxide (8.00 ml). After stirring at room temperature for 0.5 h and then the temperature was raised to 85 °C and then 10% sodium hydroxide (8.00 ml) and *N*-benzyl-2-chloroacetamide (2.20 g, 12.0 mmol) were added. The reaction mixture was stirred under reflux until complete conversion of the starting materials (6 h, monitored by TLC). The mixture was then cooled to room temperature and crystallized to give 3.17 g of an yellow solid (84% yield).The product was recrystallized from ethanol to afford the title compound as an off-yellow solid (yield: 60%). Crystals suitable for X-ray analysis were obtained by slow evaporation using dichloromethane methanol (2:1 *v*/*v*) as eluent.

### Refinement

H atoms of the amino group were located in a difference map and refined freely. The remaining H atoms were positioned geometrically (C—H = 0.93–0.97 Å, N—H = 0.82–0.90 Å) and refined using a riding model, with *U*_iso_(H) = 1.2*U*_eq_(C).

### Crystal data

**Table:**

C_21_H_16_FN_3_OS	*F*(000) = 784
*M**_r_* = 377.43	*D*_x_ = 1.417 Mg m^−^^3^
Monoclinic, *P*2_1_/*c*	Mo *K*α radiation, λ = 0.7107 Å
*a* = 18.9008 (7) Å	Cell parameters from 2427 reflections
*b* = 9.9828 (4) Å	θ = 3.0–29.2°
*c* = 9.5924 (4) Å	µ = 0.21 mm^−^^1^
β = 102.224 (4)°	*T* = 293 K
*V* = 1768.89 (11) Å^3^	Block, yellow
*Z* = 4	0.40 × 0.30 × 0.10 mm

### Data collection

**Table:**

Oxford Diffraction Xcalibur Eos diffractometer	3609 independent reflections
Radiation source: Enhance (Mo) X-ray Source	2665 reflections with *I* > 2σ(*I*)
Graphite monochromator	*R*_int_ = 0.021
Detector resolution: 16.0874 pixels mm^-1^	θ_max_ = 26.4°, θ_min_ = 3.0°
ω scans	*h* = −21→23
Absorption correction: multi-scan (*CrysAlis PRO*; Oxford Diffraction, 2006)	*k* = −12→11
*T*_min_ = 0.991, *T*_max_ = 1.000	*l* = −11→11
7919 measured reflections	

### Refinement

**Table:**

Refinement on *F*^2^	Primary atom site location: structure-invariant direct methods
Least-squares matrix: full	Secondary atom site location: difference Fourier map
*R*[*F*^2^ > 2σ(*F*^2^)] = 0.044	Hydrogen site location: inferred from neighbouring sites
*wR*(*F*^2^) = 0.104	H atoms treated by a mixture of independent and constrained refinement
*S* = 1.03	*w* = 1/[σ^2^(*F*_o_^2^) + (0.0422*P*)^2^ + 0.2875*P*] where *P* = (*F*_o_^2^ + 2*F*_c_^2^)/3
3609 reflections	(Δ/σ)_max_ < 0.001
252 parameters	Δρ_max_ = 0.23 e Å^−^^3^
0 restraints	Δρ_min_ = −0.23 e Å^−^^3^

### Special details

**Table:**

Geometry. All e.s.d.'s (except the e.s.d. in the dihedral angle between two l.s. planes) are estimated using the full covariance matrix. The cell e.s.d.'s are taken into account individually in the estimation of e.s.d.'s in distances, angles and torsion angles; correlations between e.s.d.'s in cell parameters are only used when they are defined by crystal symmetry. An approximate (isotropic) treatment of cell e.s.d.'s is used for estimating e.s.d.'s involving l.s. planes.
Refinement. Refinement of *F*^2^ against ALL reflections. The weighted *R*-factor *wR* and goodness of fit *S* are based on *F*^2^, conventional *R*-factors *R* are based on *F*, with *F* set to zero for negative *F*^2^. The threshold expression of *F*^2^ > σ(*F*^2^) is used only for calculating *R*-factors(gt) *etc*. and is not relevant to the choice of reflections for refinement. *R*-factors based on *F*^2^ are statistically about twice as large as those based on *F*, and *R*- factors based on ALL data will be even larger.

### Fractional atomic coordinates and isotropic or equivalent isotropic displacement parameters (Å^2^)

**Table:**

	*x*	*y*	*z*	*U*_iso_*/*U*_eq_	
S1	0.20491 (3)	0.22083 (5)	0.33501 (5)	0.04353 (16)	
F1	0.68007 (7)	0.14850 (17)	0.54263 (17)	0.0879 (5)	
O1	0.01663 (7)	0.12650 (13)	0.08498 (13)	0.0415 (3)	
N1	0.34252 (8)	0.16214 (16)	0.32473 (17)	0.0430 (4)	
N2	0.12841 (11)	0.01149 (18)	−0.01334 (18)	0.0431 (4)	
N3	0.03925 (8)	0.26248 (15)	0.27578 (16)	0.0371 (4)	
H3	0.0698	0.2893	0.3502	0.044*	
C1	0.61019 (12)	0.1375 (3)	0.4718 (3)	0.0612 (6)	
C2	0.55976 (13)	0.2160 (3)	0.5132 (3)	0.0649 (7)	
H2	0.5730	0.2768	0.5876	0.078*	
C3	0.48814 (12)	0.2039 (2)	0.4427 (3)	0.0588 (6)	
H3A	0.4532	0.2570	0.4706	0.071*	
C4	0.46761 (11)	0.1141 (2)	0.3312 (2)	0.0467 (5)	
C5	0.52116 (12)	0.0370 (3)	0.2941 (3)	0.0629 (7)	
H5	0.5088	−0.0248	0.2204	0.075*	
C6	0.59308 (12)	0.0489 (3)	0.3640 (3)	0.0682 (7)	
H6	0.6287	−0.0031	0.3369	0.082*	
C7	0.38985 (11)	0.0995 (2)	0.2613 (2)	0.0441 (5)	
C8	0.36703 (11)	0.0228 (2)	0.1384 (2)	0.0560 (6)	
H8	0.4013	−0.0189	0.0964	0.067*	
C9	0.29486 (11)	0.0081 (2)	0.0786 (2)	0.0514 (6)	
H9	0.2799	−0.0429	−0.0035	0.062*	
C10	0.24457 (10)	0.07078 (18)	0.14290 (19)	0.0363 (4)	
C11	0.27268 (10)	0.14647 (18)	0.26477 (19)	0.0370 (4)	
C12	0.16689 (10)	0.07362 (17)	0.10565 (19)	0.0335 (4)	
C13	0.13826 (10)	0.15083 (17)	0.19911 (18)	0.0334 (4)	
C14	0.06103 (10)	0.17874 (17)	0.18296 (18)	0.0322 (4)	
C15	−0.03518 (10)	0.30762 (18)	0.2511 (2)	0.0383 (5)	
H15A	−0.0510	0.3280	0.1504	0.046*	
H15B	−0.0365	0.3905	0.3032	0.046*	
C16	−0.08885 (10)	0.21225 (17)	0.29233 (18)	0.0337 (4)	
C17	−0.16173 (11)	0.2454 (2)	0.2581 (2)	0.0465 (5)	
H17	−0.1762	0.3236	0.2073	0.056*	
C18	−0.21297 (12)	0.1646 (2)	0.2981 (3)	0.0582 (6)	
H18	−0.2615	0.1892	0.2755	0.070*	
C19	−0.19253 (12)	0.0472 (2)	0.3715 (2)	0.0540 (6)	
H19	−0.2270	−0.0075	0.3988	0.065*	
C20	−0.12068 (11)	0.0121 (2)	0.4037 (2)	0.0451 (5)	
H20	−0.1066	−0.0674	0.4521	0.054*	
C21	−0.06921 (10)	0.09380 (18)	0.36486 (19)	0.0382 (4)	
H21	−0.0207	0.0689	0.3877	0.046*	
H2A	0.1510 (12)	−0.042 (2)	−0.052 (2)	0.056 (7)*	
H2B	0.0817 (12)	−0.005 (2)	−0.013 (2)	0.050 (6)*	

### Atomic displacement parameters (Å^2^)

**Table:**

	*U*^11^	*U*^22^	*U*^33^	*U*^12^	*U*^13^	*U*^23^
S1	0.0332 (3)	0.0534 (3)	0.0409 (3)	−0.0001 (2)	0.0009 (2)	−0.0129 (2)
F1	0.0331 (7)	0.1281 (13)	0.0949 (12)	−0.0129 (8)	−0.0034 (7)	0.0030 (10)
O1	0.0347 (7)	0.0465 (8)	0.0401 (8)	−0.0024 (6)	0.0006 (6)	−0.0115 (6)
N1	0.0315 (9)	0.0477 (10)	0.0467 (10)	−0.0001 (8)	0.0010 (8)	−0.0024 (8)
N2	0.0341 (10)	0.0547 (11)	0.0402 (10)	−0.0041 (9)	0.0074 (8)	−0.0145 (8)
N3	0.0326 (9)	0.0431 (9)	0.0338 (8)	−0.0009 (7)	0.0032 (7)	−0.0076 (7)
C1	0.0297 (12)	0.0809 (17)	0.0694 (16)	−0.0101 (12)	0.0023 (11)	0.0138 (14)
C2	0.0439 (14)	0.0742 (16)	0.0707 (17)	−0.0118 (13)	−0.0009 (12)	−0.0073 (13)
C3	0.0385 (13)	0.0658 (15)	0.0693 (16)	−0.0040 (12)	0.0050 (11)	−0.0043 (12)
C4	0.0320 (11)	0.0515 (12)	0.0553 (13)	−0.0018 (10)	0.0060 (10)	0.0072 (10)
C5	0.0375 (13)	0.0783 (17)	0.0714 (17)	0.0007 (12)	0.0083 (12)	−0.0095 (13)
C6	0.0351 (13)	0.0916 (19)	0.0772 (18)	0.0046 (13)	0.0108 (12)	−0.0017 (15)
C7	0.0341 (11)	0.0488 (12)	0.0481 (12)	0.0001 (10)	0.0057 (9)	0.0025 (9)
C8	0.0366 (12)	0.0725 (15)	0.0592 (14)	0.0053 (11)	0.0109 (11)	−0.0146 (12)
C9	0.0399 (12)	0.0651 (14)	0.0478 (13)	0.0023 (11)	0.0057 (10)	−0.0162 (10)
C10	0.0332 (10)	0.0390 (10)	0.0357 (10)	0.0003 (9)	0.0049 (8)	0.0007 (8)
C11	0.0320 (10)	0.0388 (10)	0.0379 (10)	−0.0003 (9)	0.0024 (8)	0.0020 (8)
C12	0.0337 (10)	0.0339 (9)	0.0317 (10)	−0.0023 (8)	0.0040 (8)	0.0024 (8)
C13	0.0319 (10)	0.0345 (10)	0.0318 (9)	−0.0022 (8)	0.0022 (8)	−0.0003 (8)
C14	0.0336 (10)	0.0316 (9)	0.0301 (9)	−0.0017 (8)	0.0040 (8)	0.0028 (7)
C15	0.0375 (11)	0.0389 (10)	0.0375 (11)	0.0074 (9)	0.0058 (9)	−0.0005 (8)
C16	0.0346 (10)	0.0358 (10)	0.0298 (9)	0.0027 (9)	0.0046 (8)	−0.0074 (7)
C17	0.0375 (12)	0.0476 (12)	0.0527 (13)	0.0085 (10)	0.0060 (10)	0.0030 (9)
C18	0.0320 (12)	0.0698 (15)	0.0725 (16)	0.0062 (12)	0.0104 (11)	−0.0006 (13)
C19	0.0427 (13)	0.0606 (14)	0.0607 (14)	−0.0075 (11)	0.0157 (11)	−0.0009 (11)
C20	0.0473 (13)	0.0445 (11)	0.0435 (12)	−0.0018 (10)	0.0094 (10)	0.0000 (9)
C21	0.0339 (11)	0.0447 (11)	0.0349 (10)	0.0043 (9)	0.0045 (8)	−0.0008 (8)

### Geometric parameters (Å, º)

**Table:**

S1—C11	1.7353 (19)	C7—C8	1.394 (3)
S1—C13	1.7548 (18)	C8—H8	0.9300
F1—C1	1.356 (2)	C8—C9	1.371 (3)
O1—C14	1.235 (2)	C9—H9	0.9300
N1—C7	1.339 (2)	C9—C10	1.387 (3)
N1—C11	1.333 (2)	C10—C11	1.399 (2)
N2—C12	1.366 (2)	C10—C12	1.436 (3)
N2—H2A	0.82 (2)	C12—C13	1.377 (2)
N2—H2B	0.90 (2)	C13—C14	1.462 (2)
N3—H3	0.8600	C15—H15A	0.9700
N3—C14	1.348 (2)	C15—H15B	0.9700
N3—C15	1.448 (2)	C15—C16	1.504 (3)
C1—C2	1.357 (3)	C16—C17	1.387 (3)
C1—C6	1.347 (3)	C16—C21	1.382 (2)
C2—H2	0.9300	C17—H17	0.9300
C2—C3	1.384 (3)	C17—C18	1.376 (3)
C3—H3A	0.9300	C18—H18	0.9300
C3—C4	1.386 (3)	C18—C19	1.379 (3)
C4—C5	1.377 (3)	C19—H19	0.9300
C4—C7	1.488 (3)	C19—C20	1.373 (3)
C5—H5	0.9300	C20—H20	0.9300
C5—C6	1.388 (3)	C20—C21	1.380 (3)
C6—H6	0.9300	C21—H21	0.9300
			
C11—S1—C13	90.80 (9)	C11—C10—C12	112.62 (16)
C11—N1—C7	116.41 (17)	N1—C11—S1	121.82 (15)
C12—N2—H2A	116.1 (16)	N1—C11—C10	126.18 (18)
C12—N2—H2B	115.6 (13)	C10—C11—S1	112.00 (14)
H2A—N2—H2B	119 (2)	N2—C12—C10	122.31 (17)
C14—N3—H3	119.8	N2—C12—C13	125.82 (18)
C14—N3—C15	120.47 (15)	C13—C12—C10	111.78 (16)
C15—N3—H3	119.8	C12—C13—S1	112.79 (14)
F1—C1—C2	118.5 (2)	C12—C13—C14	123.99 (16)
C6—C1—F1	119.2 (2)	C14—C13—S1	123.12 (13)
C6—C1—C2	122.3 (2)	O1—C14—N3	120.75 (17)
C1—C2—H2	120.6	O1—C14—C13	120.16 (16)
C1—C2—C3	118.8 (2)	N3—C14—C13	119.09 (16)
C3—C2—H2	120.6	N3—C15—H15A	108.2
C2—C3—H3A	119.4	N3—C15—H15B	108.2
C2—C3—C4	121.2 (2)	N3—C15—C16	116.49 (15)
C4—C3—H3A	119.4	H15A—C15—H15B	107.3
C3—C4—C7	119.98 (19)	C16—C15—H15A	108.2
C5—C4—C3	117.4 (2)	C16—C15—H15B	108.2
C5—C4—C7	122.6 (2)	C17—C16—C15	118.56 (16)
C4—C5—H5	119.1	C21—C16—C15	123.34 (17)
C4—C5—C6	121.7 (2)	C21—C16—C17	118.10 (18)
C6—C5—H5	119.1	C16—C17—H17	119.5
C1—C6—C5	118.6 (2)	C18—C17—C16	121.10 (19)
C1—C6—H6	120.7	C18—C17—H17	119.5
C5—C6—H6	120.7	C17—C18—H18	119.9
N1—C7—C4	116.12 (18)	C17—C18—C19	120.2 (2)
N1—C7—C8	121.59 (18)	C19—C18—H18	119.9
C8—C7—C4	122.28 (19)	C18—C19—H19	120.4
C7—C8—H8	119.5	C20—C19—C18	119.2 (2)
C9—C8—C7	121.0 (2)	C20—C19—H19	120.4
C9—C8—H8	119.5	C19—C20—H20	119.7
C8—C9—H9	120.6	C19—C20—C21	120.6 (2)
C8—C9—C10	118.70 (19)	C21—C20—H20	119.7
C10—C9—H9	120.6	C16—C21—H21	119.6
C9—C10—C11	116.14 (18)	C20—C21—C16	120.79 (18)
C9—C10—C12	131.23 (18)	C20—C21—H21	119.6
			
S1—C13—C14—O1	179.94 (13)	C9—C10—C11—N1	−0.9 (3)
S1—C13—C14—N3	0.3 (2)	C9—C10—C12—N2	−2.3 (3)
F1—C1—C2—C3	−179.1 (2)	C9—C10—C12—C13	−179.1 (2)
F1—C1—C6—C5	178.8 (2)	C10—C12—C13—S1	−0.40 (19)
N1—C7—C8—C9	−0.5 (3)	C10—C12—C13—C14	176.11 (16)
N2—C12—C13—S1	−177.12 (15)	C11—S1—C13—C12	0.46 (14)
N2—C12—C13—C14	−0.6 (3)	C11—S1—C13—C14	−176.09 (15)
N3—C15—C16—C17	−173.84 (16)	C11—N1—C7—C4	−178.27 (17)
N3—C15—C16—C21	7.2 (2)	C11—N1—C7—C8	0.5 (3)
C1—C2—C3—C4	−0.3 (4)	C11—C10—C12—N2	176.96 (16)
C2—C1—C6—C5	−0.6 (4)	C11—C10—C12—C13	0.1 (2)
C2—C3—C4—C5	0.4 (3)	C12—C10—C11—S1	0.2 (2)
C2—C3—C4—C7	177.6 (2)	C12—C10—C11—N1	179.78 (17)
C3—C4—C5—C6	−0.7 (4)	C12—C13—C14—O1	3.8 (3)
C3—C4—C7—N1	−9.0 (3)	C12—C13—C14—N3	−175.88 (16)
C3—C4—C7—C8	172.3 (2)	C13—S1—C11—N1	−179.96 (16)
C4—C5—C6—C1	0.8 (4)	C13—S1—C11—C10	−0.39 (14)
C4—C7—C8—C9	178.2 (2)	C14—N3—C15—C16	80.5 (2)
C5—C4—C7—N1	167.95 (19)	C15—N3—C14—O1	−8.7 (3)
C5—C4—C7—C8	−10.8 (3)	C15—N3—C14—C13	170.97 (15)
C6—C1—C2—C3	0.4 (4)	C15—C16—C17—C18	−177.43 (18)
C7—N1—C11—S1	179.73 (14)	C15—C16—C21—C20	178.02 (17)
C7—N1—C11—C10	0.2 (3)	C16—C17—C18—C19	−1.0 (3)
C7—C4—C5—C6	−177.7 (2)	C17—C16—C21—C20	−0.9 (3)
C7—C8—C9—C10	−0.2 (3)	C17—C18—C19—C20	−0.2 (3)
C8—C9—C10—C11	0.8 (3)	C18—C19—C20—C21	0.8 (3)
C8—C9—C10—C12	180.0 (2)	C19—C20—C21—C16	−0.3 (3)
C9—C10—C11—S1	179.59 (15)	C21—C16—C17—C18	1.5 (3)

### Hydrogen-bond geometry (Å, º)

**Table:**

*D*—H···*A*	*D*—H	H···*A*	*D*···*A*	*D*—H···*A*
N3—H3···S1	0.86	2.68	3.0897 (16)	111
N3—H3···N2^i^	0.86	2.51	3.253 (2)	146
N2—H2*B*···O1	0.90 (2)	2.15 (2)	2.741 (2)	122.7 (17)
N2—H2*B*···O1^ii^	0.90 (2)	2.20 (2)	3.015 (2)	150.0 (17)

Symmetry codes: (i) *x*, −*y*+1/2, *z*+1/2; (ii) −*x*, −*y*, −*z*.
